# Postnatal Systemic Blood Flow in Neonates with Abnormal Fetal Umbilical Artery Doppler

**DOI:** 10.1155/2014/957180

**Published:** 2014-03-05

**Authors:** Richelle N. Olsen, Jennifer Shepherd, Anup Katheria

**Affiliations:** ^1^Department of Reproductive Medicine, University of California, San Diego, CA, USA; ^2^Department of Neonatology, University of California, San Diego, CA, USA; ^3^Neonatal Research Institute, Sharp Mary Birch Hospital for Women and Newborns, San Diego, CA 92123, USA

## Abstract

*Objective*. Abnormal umbilical artery Doppler (UAD) studies are associated with poor neonatal outcomes. We sought to determine if postnatal measures of systemic blood flow (SBF), as measured by functional echocardiography (fECHO), could identify which fetuses with abnormal UAD were at the highest risk of adverse outcomes. *Study Design*. This is a retrospective review of fetuses with abnormal UAD who received fECHO in the first 72 hours of life. Measures of SBF (right ventricular output (RVO) and superior vena cava (SVC) flow) were performed and compared with prenatal variables and postnatal outcomes. *Result*. 63 subjects had abnormal UAD, 20 of which also had fECHO. Six subjects had abnormal flow. Gestational age at delivery was similar between the two groups. Those with abnormal SBF had fewer days of abnormal UAD prior to delivery and developed RDS (*P* < 0.001). *Conclusion*. Postnatal measures of SBF were associated with poor postnatal outcomes in fetuses with abnormal UAD. Future studies incorporating antenatal measures of SBF may help obstetricians determine which pregnancies complicated by UAD are likely to have postnatal morbidity.

## 1. Introduction

Abnormal fetal umbilical artery Doppler (UAD) studies represent a problem that is complex in both antenatal prevention and management and postnatal management [1]. In particular, absent and reversed end-diastolic flow of the fetal umbilical arteries are associated with poor neonatal outcomes, ranging from premature delivery and stillbirth to postnatal neurodevelopmental impairment [2] and diseases such as obesity and hypertension later in life [2]. Reversed end-diastolic flow (REDF) is the most advanced stage of abnormal umbilical artery Doppler flow and represents obliteration of nearly 70% of the placental function [3]. REDF also represents a higher risk of NICU admission, need for respiratory support, and perinatal mortality, regardless of age at delivery [4].

As placental function declines, the changes noted in fetal venous Doppler studies represent major changes in the fetal circulation in response to hypoxia. The increase in placental resistance leads to an obliteration of small muscular placental arteries, which leads to a decrease in the diastolic flow in the umbilical artery Doppler. The fetus responds with an increase in red blood cell mass and shunting of blood to nonessential vascular beds in order to increase oxygen utilization [5, 6]. This results in preferential cardiac and cerebral blood flow, with reduced blood flow to the rest of the body [7, 8]. As this process continues, the fetal right ventricular afterload increases in the setting of myocardial impairment, and changes in the fetal ductus venosus can often be visualized as a late and ominous finding [9].

Many studies have attempted to elucidate the factors that most strongly predict perinatal outcomes after delivery in the setting of abnormal UAD; however to date gestational age has always been most predictive [10]. More recently postnatal hemodynamic evaluation of preterm neonates has become part of the routine assessment in many European and Australian centers. Postnatal functional echocardiography (fECHO) uses measures of systemic blood flow (SBF) that have been shown to be more predictive than traditional measures of perfusion such as heart rate and blood pressure monitoring for poor outcomes. Infants identified to have low SBF, as indicated either by low superior vena cava flow (SVC) or low right ventricular output (RVO), have a higher risk of mortality and morbidity such as intraventricular hemorrhage [11, 12]. Given the high rate of poor neonatal outcomes in the setting of abnormal fetal UAD and low SBF, we sought to identify which antenatal factors could predict low SBF in pregnancies complicated by abnormal UAD.

## 2. Materials and Methods

This is a retrospective review of fetuses who are delivered prematurely in the setting of abnormal UAD who received a fECHO in the first 72 hours. IRB approval for the study was obtained from our institution. A list of all fetuses with abnormal Doppler studies that were cared for and delivered at the University of California, San Diego, between August 2008 and April of 2012 was collected into a database.

Antenatal variables identified and collected from the electronic charts were gestational age at delivery, gravity and parity, ethnicity, chorionicity, maternal age at delivery, gestational age at the time of initial abnormal Doppler studies, number of days from initial identification of abnormal UAD until delivery, administration of maternal steroids, estimated fetal weight percentile prior to delivery, last measured amniotic fluid index (AFI), maternal BMI, maternal disease (including diabetes, hypertension, preeclampsia, and abruption), indication for delivery, and mode of delivery. Prenatal ultrasound data collected for each delivery included gestational age at first abnormal Doppler flow (defined as absent or reversed end-diastolic flow in the umbilical artery), the number of days of abnormal Doppler flow prior to delivery, and the presence of any other Doppler flow abnormalities at the time of delivery (such as abnormal ductus venosus flow or middle cerebral artery abnormalities).

Postnatal clinical variables collected were birth weight and birth weight percentile, APGAR scores, gender, presence of congenital anomalies, number of hospital days, death prior to discharge, presence of respiratory distress syndrome (RDS), presence of intraventricular hemorrhage (IVH), and placental pathology. Postnatal fECHO measurements collected were SVC flow and RVO. A low SVC flow was defined as <50 mL/kg/min and a low RVO was defined as <150 mL/kg/min.

**Figure 1:**
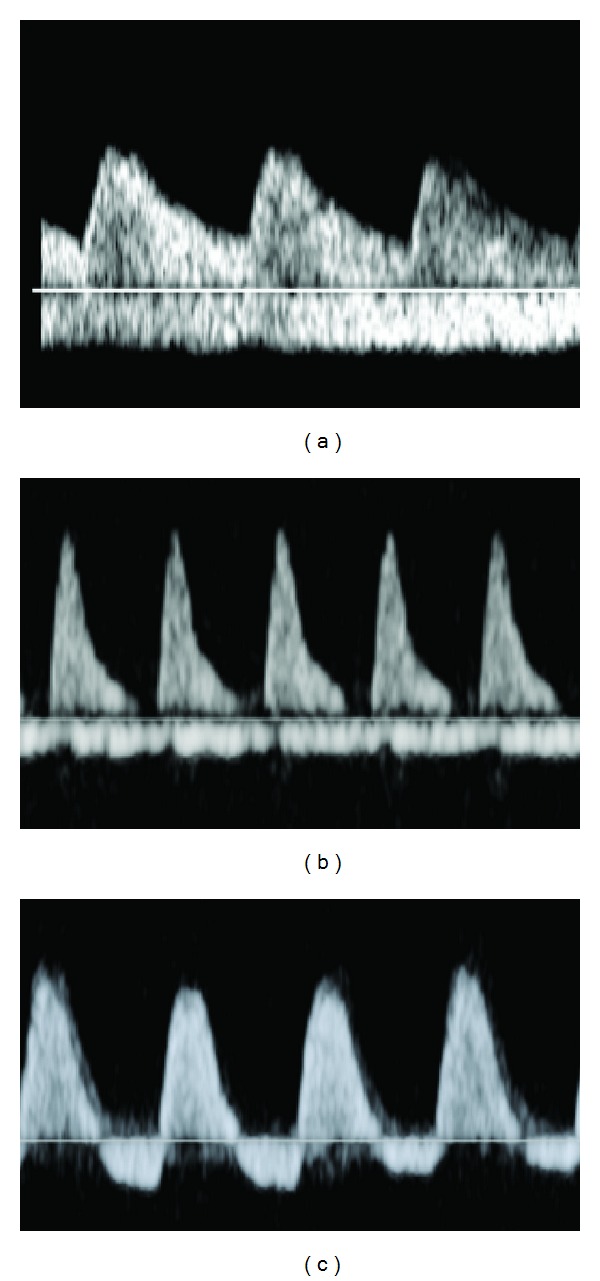
Umbilical artery Doppler studies. Examples of (a) normal, (b) absent, and (c) reversed end-diastolic flow.

Patients were included in the study if they had both abnormal antenatal UAD studies and a postnatal echocardiogram within the first 72 hours of life. Pregnancies were excluded from the study if the fetuses were known to have congenital anomalies or any heart defect other than a patent ductus arteriosus, or a small ventral septal defect. Indication for evaluation with Doppler studies was at the discretion of the provider; however, common indications included suspected growth abnormalities, abnormal fluid levels, or previously documented Doppler abnormalities. All pregnant patients were scanned with a General Electric E8 ultrasound (GE Medical Systems, Milwaukee, WI, USA) by either a perinatologist or sonographer with advanced fetal sonography training, and umbilical artery Doppler velocimetry waveforms were obtained in the midportion of the cord during periods of fetal inactivity without breathing being present (see Figure 1).

Postnatal functional echocardiograms were performed when a trained provider in echocardiography was available and/or there was a clinical indication. Prematurity, hypotension, clinical instability, and evaluation for patent ductus arteriosus (PDA) were common clinical indications for fECHO in the first 72 hours of life. fECHO was performed and interpreted at the bedside by neonatologists trained in echocardiography using the General Electric Vivid E9 cardiovascular ultrasound system (GE Medical Systems, Milwaukee, WI, USA) with either the 7S or 10S phased array transducer probe. Original recorded measures for SBF without knowledge of the antenatal Dopplers were used for purposes of minimizing any bias for the study.

SVC flow was calculated from the vessel diameter obtained in the parasternal long axis window in a sagittal plane and from the velocity obtained in the subcostal window. The average SVC diameter was obtained by measuring the maximum and minimum diameters at the junction of the SVC and right atrium over three cardiac cycles and all 6 measurements averaged. The complete velocity time integral from 10 consecutive cardiac cycles displaying laminar flow was obtained and averaged. SVC flow was calculated by measuring the average velocity time integral and multiplying it by the average cross-sectional area of the superior vena cava (mm) and the heart rate (beats per minute).

**Figure 2:**
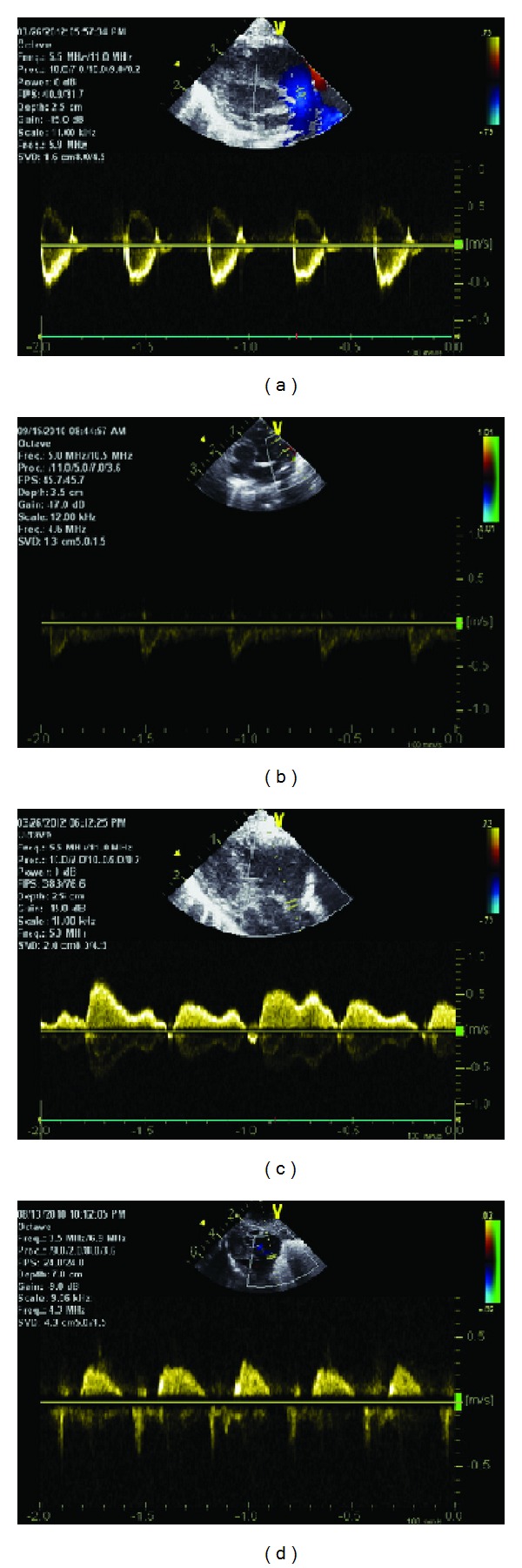
fECHO—normal SVC and RO measurements. Examples of (a) normal RO flow, (b) low (abnormal) RO flow, (c) normal SVC flow, and (d) low (abnormal) SVC flow.

Right ventricular output (RVO) was obtained by imaging the pulmonary artery from the parasternal long axis window in the sagittal plane to obtain both the vessel diameter and the velocity. The complete velocity time integral from 5 consecutive cardiac cycles displaying laminar flow was obtained and averaged. RVO was calculated by multiplying the velocity time integral by the cross-sectional area of the pulmonary artery (cm) and the heart rate (beats per minute) (see Figure 2).

Descriptive statistics were performed using Student's *t*-test and Mann-Whitney *U* tests (when nonparametric data was present), along with chi square analysis for categorical outcomes. Multivariate logistic regression was used to determine independent variables associated with low SBF including maternal age, gestational age or birth percentile at delivery, and length of stay in the NICU.

## 3. Results

63 subjects were identified with abnormal uterine artery Doppler studies; 20 subjects had both abnormal UAD and fECHO performed within the first 72 hours of life. Six infants had abnormal fECHO defined as either low RVO (<150 mL/kg/min) or low SVC flow (<50 mL/kg/min). The maternal demographics were overall similar between the two groups with the exception of age, which was lower in the abnormal fECHO group (Table 1).

The gestational age at delivery was similar between the two groups. Those with abnormal fECHO had fewer days of abnormal UAD prior to delivery and trended towards a greater length of NICU stay (*P* value). The indications for deliveries were similar between the two groups as were the amniotic fluid indices at time of delivery and modes of delivery (Table 2).

Infants with abnormal fECHO had higher birth weight percentiles than those with normal fECHO and universally developed RDS. Due to the small frequency of more morbid neonatal outcomes (such as NEC, IVH, and pulmonary hemorrhage), the risk of these outcomes was not calculated (Table 3). The individual outcomes of infants with low systemic blood flow are shown in Table 4. The use of multivariate logistic regression did not significantly change the statistical significance of any of the above variables.

## 4. Discussion

The timing of when to deliver a fetus with abnormal UAD has long been challenging. The goal of delivering as mature a fetus as possible has to be balanced with the desire to minimize poor neural outcomes due to significant hypoxemia, or even death. Baschat advocated prolongation of pregnancy to 34 weeks whenever possible, due to the significant morbidities associated with preterm delivery [10]. While some suggest delivering only when either an abnormal BPP is noted or ductus venosus a-wave reversal occurs, other studies have only recommended delivery prolongation to 28 weeks with an attempt to deliver *prior* to development of cardiac decompensation [5]. The challenge with many of these studies is the correlation between prenatal cardiac function and postnatal hemodynamics.

This is the first study to describe an association between abnormal UAD and low SBF as an attempt to identify the highest risk infants. Of interest, lower postnatal SBF (abnormal SVC or RVO) was associated with a shorter duration of time from the first abnormal UAD until delivery. The factors indicating the need for imminent delivery, such as the severity of the UAD or the fetal tracings at the time of delivery, were similar between the two groups. Infants who had lower SBF were more immature, suggesting that delaying delivery to allow for more maturity was likely outweighed by other acute factors driving the decision to deliver. It is possible that the short duration abnormal Doppler studies prior to delivery were indicative of a more acute and severe underlying process, which gave insufficient time to allow a normal fetal adaptive response.

While low SBF has been shown to correlate with adverse outcomes such as death and IVH [12, 13], infants in our study with abnormal UAD as well as low SBF were at much higher risk of needing surfactant and mechanical ventilation due to RDS. While RDS is primarily directly related to the degree of prematurity, there was no significant difference in gestational age between groups that could explain the difference in rates of RDS. This similarly could be related to either acutely impaired transitional hemodynamics causing abnormal pulmonary blood flow or a short duration of fetal stress limiting the time allowed for a fetal adaptive response. While this study was not large enough to evaluate the risk of more severe neonatal morbidities (such as NEC, pulmonary, or intraventricular hemorrhage), prior studies have already demonstrated these associations [12–14].

There are several limitations to our study. Our study had a small sample size, due in part to the limited number of abnormal scans plus a neonatal provider who had performed a postnatal echocardiogram. Often, infants who had abnormal UAD who were more mature did not warrant an echocardiogram because of their stability. We also did not include infants who had normal UAD as a third control group. Unfortunately, these infants could have a number of confounding variables for both antenatal causes of delivery and reasons for postnatal low SBF. Future prospective studies should control for premature infants without abnormal UAD and similarly perform time scans to minimize changes that may occur with adaptation. Our study suggests that if antenatal measures of systemic blood flow such as SVC flow could be performed at the time of Doppler measurements of umbilical flow, this could help determine the degree of fetal impairment. These measures need further prospective evaluation.

In conclusion, our findings suggest that other measures of SBF may be a useful tool in the assessment of fetuses with abnormal UAD and may be helpful in identifying the most at risk infants in this subset of patients.

## Figures and Tables

**Table 1:** Maternal demographics.

	Normal fECHO	Abnormal fECHO	*P* value
	(*n* = 14)	(*n* = 6)	
Age	33.4 ± 5.5	27.2 ± 2.5	**0.003**
BMI	27.6 ± 4.3	31 ± 2.4	0.08
Prior term deliveries			0.142
Prior preterm deliveries			0.143
Race			0.607
Caucasian	4	3	
Hispanic	7	3	
Asian/Pacific Islander	1	0	
Other/unknown	2	0	
Preeclampsia	5	2	0.919
Abnormal serum analytes	7	1	0.347

**Table 2:** Antenatal variables.

	Normal fECHO	Abnormal fECHO	*P* value
	(*n* = 14)	(*n* = 6)	
Gestational age at first abnormal Doppler study (weeks)	26.8 ± 4.3	27.3 ± 3.5	0.794
Gestational age at delivery (weeks)	29.9 ± 2.9	27.9 ± 3.3	0.184
Duration of abnormal UAD prior to delivery (days)	23 ± 28	4.5 ± 4	**0.03**
Indication for delivery			0.239
Labor/nonspecified	0	1	
Fetal distress	4	3	
IUGR/oligohydramnios	8	2	
Preeclampsia	2	0	
Cesarean delivery	12	6	0.329

**Table 3:** Neonatal outcomes.

	Normal fECHO	Abnormal fECHO	*P* value
	(*n* = 14)	(*n* = 6)	
RVO flow (mL/kg/min)	241 ± 59	118 ± 52	**<0.001**
SVC flow (mL/kg/min)	91 ± 27	38 ± 12	**<0.001**
Male infant	8 (57%)	3 (50%)	0.636
1 minute Apgar scores <7	9 (64%)	5 (83%)	0.394
5 minute Apgar scores <7	2 (14%)	0	0.329
Length of NICU stay (days)	63 ± 38	97 ± 52	0.173
Birth weight percentile	5 ± 5	25 ± 24	**0.009**
Small for gestational age (%)	**14 (100%)**	**2 (33%)**	**<0.001**
RDS (%)	**5 (36%)**	**6 (100%)**	**0.008**
IVH	2 (14%)	2 (33%)	0.329

**Table 4:** Neonates with abnormal fECHO.

Subj. number	Number of days UAD prior to delivery (days)	GA at delivery (weeks)	Weight percentile at delivery	SVC flow (mL/kg/min)	RVO (mL/kg/min)	NICU length of stay (days)	ARDS	Intubated	IVH	Demise
1	9	25 4/7	2%	47	213	147	Yes	Yes	No	No
2	10	25 1/7	25%	29	79	NA^1^	Yes	Yes	Grade IV	Yes
3	1	25 5/7	16%	nc^2^	64	152	Yes	Yes	Grade I	No
4	4	32 6/7	11%	47	127	29	Yes	No	No	No
5	3	27	70%	21	105	87	Yes	No	No	No
6	0	31	25%	44	123	69	Yes	No	No	No

^1^Demise on day of life 2.

^2^Not calculable.
